# Supplementary material for: Are carbon emissions trading and green financial instruments synergistic? -Comprehensive quantitative research based on content analysis
Source: PLoS One. 2024 Mar 7;19(3):e0298601. doi: 10.1371/journal.pone.0298601 (PMC10919601; doi:10.1371/journal.pone.0298601)
Supplement: S1 Data — (ZIP) [file pone.0298601.s001.zip › Data/地级市Z_F工作报告.zip!/┴╔─■/╔≥╤⌠╩╨/└┤╫╘╣1⁄2╓┌║┼í╛┬φ┐╦╩2╛▌═°í┐_2012.docx]

各位代表：

　　现在，我代表市政府向大会报告工作，请予审议，并请市政协各位委员提出意见和建议。

　　一、2011年工作回顾

　　过去一年，在市委的正确领导下，在市人大及其常委会依法监督和市政协民主监督下，市政府团结带领全市人民，深入贯彻落实科学发展观，扎实推进“抓项目促调整，抓环境促提升，抓民生促和谐”等重点工作，全面完成了市十四届人大四次会议确定的各项任务，实现了“十二五”良好开局。

　　——全市经济平稳较快发展。预计地区生产总值实现5950亿元，比上年增长12.6%；规模以上工业增加值完成2900亿元，增长16%；地方财政一般预算收入完成620亿元，增长33.2%；固定资产投资完成4380亿元，增长24.1%；社会消费品零售总额实现2417亿元，增长17%。

　　——结构调整步伐不断加快。规模以上装备制造业增加值增长16.8%，快于工业增速0.8个百分点；现代服务业增加值增长11.5%，快于服务业增速0.5个百分点；“一市三县”地区生产总值增长24.8%，快于全市增速12.2个百分点。

　　——城乡环境面貌持续改善。完成城建投资480亿元，增长20%。道路交通、电力能源、生态环保等一批重大基础设施加快推进，新城建设、老城改造同步提速。铁西区荣获“中国人居环境范例奖”，9个涉农区县（市）、开发区全部通过国家生态区县验收或技术核查。全市单位地区生产总值能耗下降3.77%，化学需氧量、二氧化硫排放总量分别下降2.5%和1.5%。

　　——人民生活水平稳步提高。教育、社保、医疗等民生支出达到281亿元，增长24.9%。城市居民人均可支配收入达到23320元，增长13.5%；农民人均纯收入达到11575元，增长15.5%。城镇登记失业率为3%，养老保险实现城乡全覆盖，民生工程10件实事全面完成。

　　一年来，我们主要抓了以下几项工作。

　　（一）加强空间和项目建设，努力保持强劲发展势头

　　着力做优发展空间，切实加强项目建设，为经济持续快速增长注入强大动力。

　　空间建设向纵深推进。行政区划调整的政策效应进一步显现，各个区域竞相发展。浑南新城建设步伐加快，金廊工程全面提速，园区基础设施、配套设施日趋完善，对产业的承载力和项目的吸引力明显增强。沈阳经济区城际连接带建设全面推进，19个新城新市镇成为吸引各方投资的新空间。

　　项目建设取得新进展。全市开（复）工建设投资3000万元以上项目2206个，完成投资3945.3亿元，分别增长15.3%和69.1%；其中10亿元以上项目332个，完成投资2235.8亿元，分别增长31.7%和88.9%。260个重点项目和60个政府投资类项目进展良好。土地、资金等要素保障有力，一批对结构调整具有较强牵动力的重大项目加快建设，为沈阳当前和长远发展提供了有力支撑。

　　（二）加大结构调整力度，进一步提升经济综合实力

　　坚持以增量调整经济结构，以创新促进产业升级，推动经济发展速度、质量、效益同步提升。

　　增强工业经济实力。深入实施工业“五项工程”，加快装备制造业“三大聚集区”和“两大配套区”建设，规模以上工业总产值迈上万亿元台阶，达到1.16万亿元，增长25.8%。主营业务收入超百亿元企业达到8户，机床集团销售收入跃升为全世界同行业首位。上通北盛（三期）、沃得产业园、海尔工业园、希杰生化等一批重大项目开工建设，米其林轮胎、三一重装综采成套设备等项目加快推进，华晨宝马新工厂、上通北盛商务车升级改造等项目实现投产。完成国内外并购项目17个。汽车及零部件产业集群实现千亿元产出，达到1030亿元。28个重点产业集群完成产值5055亿元，增长30%。

　　提升服务业发展水平。服务业增加值增长11%。太原街都市商贸中心、北站金融商贸区等5个集聚区营业收入均突破百亿元。方特欢乐世界开园营业，华润万象城、龙之梦亚太城等大型城市综合体投入运营。华汇人寿保险、民生银行获准筹建，沈阳农村商业银行正式营业，全市分行分公司以上金融机构达到110家。沈阳国际物流港、沈北综合物流园、沈西制造业物流园等物流园区加快建设。成功举办制博会、糖酒会、广告节、农博会等各类展会，会展交易额增长12.8%，我市荣获“中国十佳品牌会展城市”称号。温泉旅游成为旅游业新亮点，棋盘山成为省级旅游集聚区，中国沈阳国际旅游节影响力进一步提升，全市旅游总收入达到663.3亿元，增长28.3%，接待国内外游客6200万人次。房地产业平稳健康发展。

　　推动县域快速发展。认真落实强农惠农政策，促进农业发展、农民增收、农村和谐。农田水利建设投入增长8倍，有力改善了农业生产条件。全市粮食总产量达到40.6亿公斤，再创历史新高。新增设施农业和高效特色农业面积37.7万亩，新增“一乡一业”专业乡镇12个，开工建设规模以上农业产业化项目120个，新增农民专业合作社924个，培育农村产业带头人500名。新民玖龙纸业、辽中铸锻造、法库伊奈建筑、康平碳纤维等重大项目加快推进，县域重点产业集群中有11个产值超百亿元。出台支持县域经济发展政策措施，“一市三县”综合实力明显提升。

　　积极培育新兴产业。建立物联网、机器人等产业技术创新联盟，新增2家国家重点实验室。IC装备、高档数控机床等重大科技专项实现新突破。沈鼓研制的20兆瓦电驱压缩机填补国内空白。我市成为全国第一个现代建筑产业化试点城市。国家大学科技城、泗水科技城、航高基地、手机产业园等重点园区加快建设，国际特种机床装备城成为国家级高新技术产业化基地，全市高新技术产品增加值完成1201亿元，增长19.3%。

　　（三）强化城市建设管理，大力改善城乡环境面貌

　　以建设国家中心城市为目标，抓住筹备全运会契机，全面启动新一轮大规模城市建设。

　　基础设施建设力度加大。哈大客运专线主体完工，沈阳新南站交通枢纽建设和沈阳桃仙国际机场T3航站楼改扩建进展顺利，地铁2号线通车运营，沈本产业大道、沈彰开发大道（二期）、沈阜开发大道（二期）竣工通车，一、二、三、四环同时改造建设，启动建设跨浑河大桥3座，沈阳立体化交通网络骨架正在形成。辽中500千伏输变电工程加快建设。大伙房水库输水配套工程实现送水。西气东输工程开始向沈阳供气。

　　全运会筹备工作进展顺利。浑南新城“三村三中心”及各类基础设施和配套设施加快建设，152公里道路具备通行条件，电力、热力、给水等管线铺设工程基本完成，综合管廊已完成17公里工程建设。各类场馆建设和改造工程顺利推进，市区两级全民健身中心、健身带加快建设。市场开发、安全保障、城市宣传、来宾接待等各项筹备工作有序推进。

　　生态建设迈出新步伐。辽河沈阳段治理与保护工作取得明显成效，蒲河生态廊道建设取得重大进展，百里环城水系改造加快推进。大力实施三北防护林和城市园林绿化工程，完成146块重点街路绿地景观化改造，全市植树造林59.7万亩。实施重点企业锅炉脱硫改造，建成9座污水处理厂、4座农村生活垃圾处理场。环境建设样板城创建工作扎实推进。

　　城市管理水平不断提升。完成《沈阳市综合交通规划》编制工作。开展18条城市出口路及铁路沿线等重点区域综合整治，市容环境明显改观。新增和更新公交车辆800台。加大交通管理力度，全市道路交通事故率下降18.7%。实行市政、园林、市容、环卫“四合一”城市综合监管新模式，数字化城管水平进一步提高。

　　（四）积极推进改革开放，不断增强城市发展活力

　　抓住综合配套改革启动实施的新机遇，不断拓宽改革领域，提高开放水平，努力创造加快发展的新优势。

　　继续加大改革力度。编制完成新型工业化综合配套改革试验方案，沈阳成为国家级信息化与工业化融合试验区，沈北新区、于洪区、苏家屯区分别成为省综合配套改革先导区、统筹城乡发展综合配套改革示范区和新型城镇化示范区，沈阳经济区一体化进程不断加快。继续推进国有企业改革，红梅集团完成重组，机床、沈鼓股份制改造进展顺利。支持企业上市融资，远大铝业、罕王控股在香港联交所挂牌，蓝英自动化等3户企业获批上市。土地流转、林权制度等农村改革继续深化。医药卫生体制改革三年目标全面完成。非公有制经济对全市贡献率不断提高，增加值占全市经济总量的65.7%。沈阳市中小企业服务中心成为国家中小企业公共服务示范平台。

　　不断提高开放水平。加大招商力度，9户世界500强企业在我市新设立企业11家，全市实际利用外商直接投资55亿美元，增长8.9%。沈阳综合保税区封关运行，辉山畜禽加工基地成为国家外贸转型升级示范基地，韩国周、2011国际和平日纪念活动暨东北亚发展论坛等大型涉外活动成功举办。对内招商工作取得新进展，引进投资10亿元以上项目111个，全市调入内资1210亿元，增长33%。外贸出口实现48亿美元，增长17.9%。企业“走出去”步伐加快，新签千万美元以上境外工程承包项目25个。

　　努力改善发展环境。加大软环境建设工作力度，进一步清理行政审批项目，规范行政处罚自由裁量权和行政执法检查权，推出预约服务等10项新举措，国地税实行联合办税，开通“民声微博”，开展“局长接待日”活动，便企利民服务模式不断完善。

　　（五）着力保障改善民生，促进社会更加和谐

　　始终高度关注民生，着力解决关系人民群众切身利益的实际问题，努力创造和谐稳定的良好局面。

　　积极推进就业和社会保障工作。加大创业扶持和就业培训力度，保持零就业家庭动态为零，全市新增实名制就业21.3万人，转移输出农村劳动力6.7万人。在全市企业普遍建立工资集体协商制度，集体合同签订率达到96%。全面启动新型农村养老保险和城镇居民养老保险，近40万人领取养老金。7.2万名企业老工伤人员纳入工伤保险统筹管理。保险待遇和社会救助水平进一步提高，新农合人均筹资标准由165元提高到230元。养老服务设施建设步伐加快，新建示范性日间照料站100个，新增养老床位9300张。我市荣获“全国实施妇女儿童发展纲要先进市”和“全国残疾人工作示范城市”称号。完成保障性住房5.5万套，其中公共租赁住房3.99万套。综合改造702个弃管住宅区，惠及30万户居民。新建农村饮水安全工程128处，改造213处居民供水二次加压泵站，城乡居民饮水质量得到改善。完成“节能暖房工程”200万平方米，继续实施供暖管网改造和集中供热工程，供暖质量进一步提高。

　　大力发展社会事业。加快推进教育强市、教育强区县创建工作，完成50所幼儿园改扩建、5所普通高中标准化建设和100余所学校“校安工程”，提前两年完成“双高普九”任务。深入开展贫困助学工作，市民文化素质进一步提高。开工建设市艺术中心，完成舍利塔北广场、锡伯族文化广场等一批公共文化设施改造建设，实施“二战盟军战俘营”和“审判日本战犯特别军事法庭”旧址修复。艺术惠民工程扎实推进，艺术精品生产再创佳绩，文化事业和文化产业加快发展。完成7所市属重点医院改扩建工程，群众就医环境明显改善。增设户外健身器材4000余件，建成健身场地50处，市民拥有了更多的健身设施和休闲空间。

　　努力提高社会管理水平。积极构建治安防控新体系，打击刑事犯罪成效显著，人民群众安全感不断提高。市公安局被公安部荣记“清网行动”集体一等功。开展打假专项行动，强化食品药品安全监管，有效遏制了滥用食品添加剂等违法行为。加强物价监管，完善应急机制，保障了居民生活必需品市场供应。加大安全隐患排查力度，不断完善信访“沈阳模式”，社会大局和谐稳定。

　　进一步加强民主法制工作。自觉接受市人大及其常委会的依法监督和市政协的民主监督，人大代表建议、政协提案办复率达到100%。全年提请市人大常委会审议地方性法规草案6件，发布实施政府规章11件。开展行政权力清权确权工作，强化行政执法监督和效能监察，行政权力运行更加规范。

　　国防动员、人防和民兵预备役建设进一步加强，双拥模范城创建实现“七连冠”。民族团结进步事业不断发展，对口支援工作成效显著。人口与计划生育、国家安全、保密、统计、审计、参事、宗教、侨务、贸促、广播电视、新闻出版、工商行政管理、气象、防灾减灾、档案、修志、文史、红十字会和慈善等工作都取得了新成绩。

　　各位代表，过去一年沈阳取得的成绩，是省委、省政府和市委正确领导的结果，是全市人民团结一心、奋力拼搏的结果。在此，我代表市政府，向全市各族人民致以崇高的敬意！向人大代表、政协委员，向各民主党派、工商联、无党派人士和各人民团体，向驻沈人民解放军、武警部队官兵和中、省直单位，向所有关心、支持沈阳的港澳台同胞、海外侨胞和国际友人表示衷心感谢！

　　总结过去的工作，我们也清醒地看到，我市经济社会发展中还存在一些突出的矛盾和问题。主要是经济运行质量和效益还不够高，调结构、转方式的任务繁重；与承办“十二运”特别是建设国家中心城市的要求相比，城市建设和管理水平有待进一步提升；一些关系群众切身利益的问题尚未完全解决，保障改善民生的工作力度需要进一步继续加大；按照建设人民满意的服务型政府要求，政府自身建设还需进一步加强。对此，我们要在今后工作中着力加以解决。

　　二、2012年工作安排

　　今年是我市全面实施综合配套改革的第一年，是筹备“十二运”的关键一年，也是完成本届政府任期目标的最后一年。当前，尽管国内外经济形势严峻复杂，但沈阳仍处于三大机遇叠加期。我们必须抢抓机遇、迎难而上，稳中求进、稳中求快，奋力推动沈阳在科学发展道路上迈出新步伐。

　　今年政府工作的总体要求是：深入贯彻落实市十二次党代会和市委十二届二次全会精神，以科学发展为主题，以加快转变经济发展方式为主线，紧紧围绕完成“五大任务”、实现“三大目标”、建设“实力沈阳、活力沈阳、宜居沈阳、文明沈阳、和谐沈阳”，突出抓好“深化改革、推进创新、提升文明、改善民生”等重点工作，继续保持经济社会又好又快发展，以优异成绩迎接党的十八大胜利召开。

　　综合考虑分析各方面因素，今年全市经济社会发展的主要预期目标是：地区生产总值增长11%，固定资产投资增长15%，地方财政一般预算收入增长15%，社会消费品零售总额增长15%，城市居民人均可支配收入增长11%，农民人均纯收入增长12%，城镇登记失业率控制在4%以内。

　　今年，我们要着力抓好以下几方面工作。

　　（一）保持经济平稳健康运行

　　积极应对形势变化，努力克服各种不利因素影响，及时解决经济运行中遇到的问题，保持经济增长的稳定性、协调性和可持续性。

　　强化经济运行组织协调。加强经济运行分析和监测，做好煤、电、油、运、水、气等生产要素协调保障，着力解决影响供需平衡的突出矛盾和问题。高度关注企业运行状况，支持优势骨干企业加快发展，重视和关心中小微型企业发展，进一步减轻企业负担，帮助企业解决贷款融资、技术创新、人才引进等方面困难。

　　加大项目引进建设力度。充分发挥固定资产投资对经济增长的拉动作用，加快重大产业项目、基础设施项目、新城新市镇项目、“十二运”项目、民生和社会事业项目建设，强化土地、资金等要素保障。坚持抢先抓早，围绕项目及时开（复）工，开辟绿色通道，提早做好审批及各项准备工作，确保项目按既定工期投资建设。加强对招商项目的跟踪落实，促进签约项目尽快开工、在谈项目早日落地。

　　（二）促进产业结构优化升级

　　坚持在发展中调结构，突出抓好产业集聚、产业提升、品牌塑造，着力打造领军企业，进一步增强经济综合实力。

　　努力提升制造业发展水平。继续实施工业“五项工程”，深入推进“两化”融合，提高工业经济质量和水平，规模以上工业增加值增长14%。发挥“三大聚集区”和“两大配套区”引领作用，推动装备制造业向高端化、成套化、集群化发展。支持机床、沈鼓、北方重工、远大等一批重点骨干企业加快发展、扩大规模，提高企业核心竞争力，打造世界级企业。全力抓好华晨宝马扩产、上通北盛（三期）、普利司通轮胎等汽车产业项目，加快建设重大型数控机床生产基地、富阳机床、特变电工输变电产业园等装备制造业项目，积极推进C系列飞机、Q400飞机大部件转包等航空产业项目。充分发挥重大项目和骨干企业带动作用，吸引配套企业加快集聚，进一步提高产业的集中度和丰厚度，大力培育千亿元产业集群。促进骨干企业的研发、设计、检验检测和销售集聚发展，建设铁西装备制造业大企业总部基地。大力发展新兴产业，聚焦先进装备制造、航空航天、电子信息、生物医药等重点领域，加快国家大学科技城、泗水科技城、国际特种机床装备城、现代建筑产业园等重点园区及重大项目建设，进一步壮大新兴产业规模。

　　不断增强县域经济实力。落实各项强农惠农政策，加大“三农”投入，推进县域工业化、城镇化和农业现代化。加快建设法库通航产业基地、康平高性能纤维新材料产业基地等重点园区，力争新民制药、辽中泵阀产业集群产值突破百亿元。加大农田水利等基础设施投入，实施滴灌节水农业工程，加快中低产田改造和粮食高产示范区建设，稳定粮食生产，保障主要农产品有效供给。加快农业产业化步伐，启动沈康高速公路沿线现代农业示范带建设，大力发展设施农业和高效特色农业，新增“一乡一业”专业乡镇10个，开工建设100个规模以上农业产业化项目。加快“万村千乡市场工程”和“新网工程”建设，打造专业市场和名镇名村。加快县城、新城新市镇和中心镇基础设施建设，突出抓好新民新城、辽中近海新城、法库东湖新城、康平滨湖新城建设，积极发展县域房地产业，加快城中村、城边村改造，不断提高城镇化水平。

　　加快发展服务业。扎实推进国家现代服务业综合试点，大力发展生产性服务业。支持和促进金融商贸开发区和金融街建设，努力打造东北区域金融中心。加快五洲国际工业博览城、中床国际物流、近海保税物流集中发展区等物流项目建设，促进第三方物流企业发展。继续抓好制博会、汽博会、食博会等品牌展会，引进和创建新的品牌展会。大力发展总部经济。提升生活性服务业发展水平，进一步完善中街、东中街、太原街、长江街、南塔、五爱街等商业街区服务功能。加快金廊沿线重点项目建设进度，确保全运会前金廊呈现整体形象。推进雨润农副产品全球采购中心、五洲建材城、北约克商务区等重大项目建设，新建改造一批便民市场。深度挖掘旅游资源，开发特色旅游，包装推介一批传统美食、购物名店、特色街区，努力打造旅游目的地城市。继续保持房地产业平稳健康发展。

　　大力发展文化产业。围绕建设文化强市，加快棋盘山国家文化产业示范区建设，发挥华强文化产业基地的龙头带动作用，继续引进重大文化产业项目，扶持本地文化企业做大做强。进一步发挥高校作用，推进文化科技创新，培育发展影视传媒、动漫制作、创意设计等文化产业。以清前文化、近代文化、工业文化和民族文化为重点，加强文化资源开发利用，大力推进方城、北市、西塔和西关等地区综合改造，启动北运河历史观光带建设，力争中国工业博物馆主体完工、部分开馆。

　　（三）提高城乡环境建设管理水平

　　把加强城市建设管理与筹备全运会结合起来，努力抓好基础设施和生态环境建设，进一步完善城市功能、改善城市环境、提升城市品位。

　　加快基础设施建设。围绕建设国家中心城市，着力打造大通道、建设大枢纽、构建大交通。加强沈阳经济区交通走廊建设，沈阜开发大道、沈彰开发大道按计划完工，沈抚2号公路全线竣工通车。新建、改建农村公路500公里。推进沈阳站东、西广场改造，加快沈阳新南站交通枢纽、桃仙国际机场T3航站楼建设，完成北站综合交通枢纽主体改造工程。加快一、二、三、四环改造建设进度，启动建设二、三、四环连接线，改造一批桥梁和主干道，进一步提升路网通行能力。加快地铁1、2号线延长线建设，推进地铁4、9、10号线及市区与各县（市）之间轨道交通建设的前期工作。加速浑南新城现代有轨电车建设。做好中心城区停车场、过街天桥和地下通道建设。进一步完善全市城乡电力设施规划，加快智能电网建设，努力提高电力供应能力。完善金廊配套设施，有序开发利用地下空间。

　　加强生态环境建设。全面实施“青山工程”，逐步修复山体生态环境。重点实施蒲河绿化、荒山绿化、沈西北阻沙带建设等城乡绿化工程，全市植树造林83.1万亩。实施辽河重度污染“摘帽”总攻行动，实现蒲河生态廊道三年建设目标。启动九龙河、白塔堡河、细河生态环境治理工程和“浑河西峡谷”建设，加快推进浑河两岸生态景观提升。实施百里环城水系改造，全面启动卧龙湖生态区保护与建设。科学规划建设污水处理厂，加快建设雨污分流系统，实施污泥无害化处理。进一步提高农村垃圾处理水平，建设3座垃圾处理厂和28座垃圾转运站。深入推进节能减排工作，积极开展新能源、清洁能源和再生能源开发利用。全市单位地区生产总值能耗下降3.77%，化学需氧量、二氧化硫排放总量分别下降2.5%和1.5%。继续以示范工程推动环境样板城建设，力争生态市建设通过国家技术核查。

　　提升城市科学化管理水平。加快数字化城市建设步伐，不断提升国土、规划、交通、城管、社会治安、公共服务的信息化水平。实行城市平面、立面和附属设施一体化建设和管理，大力推行公共信息标志标准化，打造一批标准化街路和精品街路。坚持公交优先发展战略，继续更新公交车辆，扩大公交网络覆盖面，密切公交与地铁线路衔接，方便市民换乘。实施机场路、三环、四环等重点街路绿化，改造北陵、南湖等10个公园，继续实施小街小巷整修工程，加大拆除违章建筑力度。大力开展市容环境专项整治，以整洁、清新、优美的城市形象迎接“十二运”。

　　做好全运会筹备工作。加快浑南新城规划道路等基础设施建设，做好华润电力浑南热电和大唐国际沈抚连接带热电项目前期工作，推进中央公园等一批公共服务设施建设，抓好绿化、水系建设和环境综合整治，确保为“十二运”配套的重点工程按计划完工。加快“三村三中心”建设，高质量完成比赛场馆、训练场馆、备用场馆新建改造工程，超前谋划体育场馆赛后利用，积极发展体育产业。加快市、区全民健身中心和健身带等公共体育设施建设，大力开展全民健身活动。制定完善交通保障、食品安全、气象服务、安全保卫等应急预案，加强实战演练。广泛开展迎全运主题教育实践活动，全面启动全国文明城市创建工作，进一步提高市民文明素质和城市文明程度。

　　（四）全面推进改革开放

　　大力推进新型工业化综合配套改革，加快构建全方位、多层次、宽领域的对外开放格局，进一步增强内在动力和发展活力。

　　努力开创改革新局面。紧紧抓住全面实施综合配套改革的重大机遇，统筹推进重点区域和关键环节的各项改革，加快构建有利于科学发展的新体制、新机制。抓好“两化”融合试点，大力推进铁西等5个试验区建设，围绕重点领域、典型企业和关键环节选取示范项目，用信息技术改造提升企业生产经营全过程，推动工业转型升级。着眼于增强自主创新能力，深入推进科技体制改革，积极发展院士工作站，建立多种形式的产业技术创新联盟，探索高新技术产业“一区多园”发展模式，加快建设国家创新型城市。深化国有企业改革，进一步扩大国有骨干企业经营自主权，积极推进重点企业股份制改造和战略重组。进一步完善区域协调发展机制，以沈抚同城化为重点，加快沈阳经济区一体化进程。

　　积极推进各项配套改革。建立健全城乡统筹发展长效机制，强化城乡基础设施有效衔接，加快公共服务、社会保障城乡均等化步伐。深入开展金融创新，积极推进国家优化金融生态综合试验，重点发展特色产业金融，促进企业与资本市场对接，加快企业上市步伐。积极探索节约集约利用土地新机制和新模式，开展促进新型工业化发展土地利用管理创新试点工作。继续深化行政管理体制改革，进一步下放行政审批权限，总结推广浑南、沈北行政管理体制机制创新经验。推进公共资源交易体系建设改革试点。推进事业单位分类改革。

　　提高对外开放水平。进一步加大招商引资力度，全市实际利用外商直接投资增长5%，调入内资增长16%。大力抓好进出口工作，加快出口产品基地建设，扩大机电产品、高新技术产品、农产品出口，积极发展服务外包产业，着力培育新的出口增长点，外贸进出口额增长10%，不断提高沈阳经济外向度。加快实施“走出去”战略，鼓励有实力的企业到国际市场竞争发展。

　　（五）着力保障改善民生

　　认真解决城乡群众最关心、最直接、最迫切的民生问题，继续为城乡群众办好事、办实事，让全市人民生活得更加幸福。

　　千方百计做好就业工作。实施更加积极的就业政策，积极开发和稳定就业岗位，加大普惠制培训力度，鼓励以创业带动就业。强化高校毕业生就业综合服务，确保困难家庭高校毕业生就业率达到100%。深入实施“双合同制”，全面开展工资集体协商，广泛开展劳动关系和谐企业创建活动。

　　不断提高社会保障水平。继续加大社会保险扩面力度，提高退休人员养老金标准，提高城镇职工和城镇居民医疗保险待遇水平，提高新型农村合作医疗筹资标准和住院报销比例，强化国有企业老工伤人员纳入工伤保险统筹后的管理和服务。完善城乡最低生活保障制度，适时调整低保标准，不断提高城乡贫困群众的救助水平。实施新一轮扶贫攻坚计划。加快市养老服务中心（二期）等大型养老机构及社区老年人日间照料站建设。不断完善残疾人保障和服务体系，提升8个区域性残疾人托养康复中心功能，推动无障碍设施进社区、进家庭。“一市三县”全部建立农村困难家庭常年病人集中康复托管服务中心。加快老旧小区改造，完成413个弃管住宅区综合改造，全面加强后续管理。全力做好住房保障工作，完成保障性住房3.68万套。继续推进集中供热和“节能暖房工程”建设，加快供暖管网改造。继续实施城市供水二次加压泵站改造工程，全面完成国家规划范围内的农村居民饮水安全工程建设。

　　加快发展社会事业。深入开展教育强市、教育强区县创建活动，推进义务教育均衡发展，新建和改扩建50所普惠制幼儿园，实施百所义务教育学校提升工程，推进普通高中标准化建设和职业教育国家试验区建设，完善终身教育体系。支持在沈高校参与地方经济社会建设。启动校车安全工程，规范农村地区校车管理。大力发展公益性文化事业，进一步完善统筹城乡的公共文化服务体系，加快市艺术中心建设，完善市图书馆、群众艺术馆等文化设施功能，推进乡镇文化站、村文化广场、农家书屋建设，继续开展艺术惠民工程。支持群众性公益服务设施建设。启动新一轮医药卫生体制改革，完善城乡医疗服务体系和公共卫生服务体系，提升县（市）中心医院、乡镇卫生院和村卫生室医疗设备水平。着力构建全民健身公共服务体系，积极开展全民健身活动。支持国防和军队现代化建设，扎实开展双拥模范城“八连冠”创建工作。

　　加强和创新社会管理。创新社区管理体制和运行机制，加快社区标准化建设，增强基层社会管理和服务能力，提升公众对社会管理的参与度。进一步强化重大事项社会稳定风险评估机制，构建多元化的社会矛盾纠纷排查调处体系，预防和化解社会矛盾。完善公共安全事件监测预警预报和快速响应机制，加强公共突发事件应急管理和设施建设。抓好“六五”普法工作，深入开展“平安沈阳”创建活动，完善社会治安防控体系。依法打击、有效防范网络犯罪，促进信息网络健康发展。强化安全生产监管，提高企业安全生产标准化水平，坚决防止重特大事故发生。进一步完善监管体系，建立健全综合协调机制，依法严厉打击制售假冒伪劣食品药品犯罪活动，保障人民群众身体健康和生命安全。

　　（六）进一步加强政府自身建设

　　越是经济形势复杂，越是工作任务繁重，越是要把政府自身建设摆到更加突出位置，抓实抓好。

　　必须严格坚持依法行政。进一步加强政府立法工作和行政执法监督，加大政务公开力度，坚持依法办事，努力建设法治政府。各级政府要自觉接受人大及其常委会的依法监督和政协的民主监督，坚持向人大报告工作和向政协通报工作制度，认真办理人大代表建议和政协提案，不断提高依法行政水平。

　　必须增强公信力和执行力。建立健全科学民主决策机制，完善重大公共政策群众参与制度，确保各项决策体现民意、符合实际。各级政府要在信用沈阳建设中发挥示范作用，一旦做出决定、决策，就必须一抓到底、实现目标，以实际行动兑现承诺、取信于民。各级领导干部要继续发扬事不过夜、事无巨细、事必躬亲的作风，把心思花在干事业上、把精力放在谋发展上、把功夫用在解难题上，直面矛盾、勇于担当，顾全大局、真抓实干。

　　必须加强廉政建设。全面推进“五大系统”建设，增强制度约束力，努力从源头上防治腐败。加强对政府重点投资项目、重点资金支出的监管和跟踪审计，严厉查处违纪违法案件。各级领导干部和全体公务员要克己奉公、廉洁自律、勤勉尽责，做人民满意的公仆。

　　各位代表，2012年是充满机遇和挑战的一年。让我们在市委的正确领导下，在市人大及其常委会依法监督和市政协民主监督下，满怀科学发展激情，坚定全面振兴信心，迎难而上、开拓奋进，全面完成今年各项工作任务，努力开创沈阳老工业基地全面振兴的新局面！
